# A Geospatial Drug Abuse Risk Assessment and Monitoring Dashboard Tailored for School Students: Development Study With Requirement Analysis and Acceptance Evaluation

**DOI:** 10.2196/48139

**Published:** 2024-07-30

**Authors:** Ahmad Mustafa Al-Aboosi, Siti Norul Huda Sheikh Abdullah, Rozmi Ismail, Khairul Nizam Abdul Maulud, Lutfun Nahar, Khairul Akram Zainol Ariffin, Meng Chun Lam, Muhamad Lazim bin Talib, Suzaily Wahab, Mahadzir Elias

**Affiliations:** 1 Faculty of Information Science & Technology Universiti Kebangsaan Malaysia Selangor Malaysia; 2 Centre for Research in Psychology and Human Well-Being Universiti Kebangsaan Malaysia Selangor Malaysia; 3 Department of Civil Engineering Universiti Kebangsaan Malaysia Selangor Malaysia; 4 School of Computing and Data Science Xiamen University Malaysia Selangor Malaysia; 5 Fakulti Sains & Teknologi Pertahanan Pertahanan Nasional Malaysia Kuala Lumpur Malaysia; 6 Department of Psychiatry Universiti Kebangsaan Malaysia Selangor Malaysia; 7 Agensi Antidadah Kebangsaan Selangor Malaysia

**Keywords:** geospatial, statistics, map, youth, drugs, dashboard, evaluation, drug abuse, monitoring, risk assessment

## Abstract

**Background:**

The enormous consequences of drugs include suicides, traffic accidents, and violence, affecting the individual, family, society, and country. Therefore, it is necessary to constantly identify and monitor the drug abuse rate among school-going youth. A geospatial dashboard is vital for the monitoring of drug abuse and related crime incidence in a decision support system.

**Objective:**

This paper mainly focuses on developing MyAsriGeo, a geospatial drug abuse risk assessment and monitoring dashboard tailored for school students. It introduces innovative functionality, seamlessly orchestrating the assessment of drug abuse usage patterns and risks using multivariate student data.

**Methods:**

A geospatial drug abuse dashboard for monitoring and analysis was designed and developed in this study based on agile methodology and prototyping. Using focus group and interviews, we first examined and gathered the requirements, feedback, and user approval of the MyAsriGeo dashboard. Experts and stakeholders such as the National Anti-Drugs Agency, police, the Federal Department of Town and Country Planning, school instructors, students, and researchers were among those who responded. A total of 20 specialists were involved in the requirement analysis and acceptance evaluation of the pilot and final version of the dashboard. The evaluation sought to identify various user acceptance aspects, such as ease of use and usefulness, for both the pilot and final versions, and 2 additional factors based on the Post-Study System Usability Questionnaire and Task-Technology Fit models were enlisted to assess the interface quality and dashboard sufficiency for the final version.

**Results:**

The MyAsriGeo geospatial dashboard was designed to meet the needs of all user types, as identified through a requirement gathering process. It includes several key functions, such as a geospatial map that shows the locations of high-risk areas for drug abuse, data on drug abuse among students, tools for assessing the risk of drug abuse in different areas, demographic information, and a self-problem test. It also includes the Alcohol, Smoking, and Substance Involvement Screening Test and its risk assessment to help users understand and interpret the results of student risk. The initial prototype and final version of the dashboard were evaluated by 20 experts, which revealed a significant improvement in the ease of use (*P*=.047) and usefulness (*P*=.02) factors and showed a high acceptance mean scores for ease of use (4.2), usefulness (4.46), interface quality (4.29), and sufficiency (4.13).

**Conclusions:**

The MyAsriGeo geospatial dashboard is useful for monitoring and analyzing drug abuse among school-going youth in Malaysia. It was developed based on the needs of various stakeholders and includes a range of functions. The dashboard was evaluated by a group of experts. Overall, the MyAsriGeo geospatial dashboard is a valuable resource for helping stakeholders understand and respond to the issue of drug abuse among youth.

## Introduction

### Background

According to the United Nations Office on Drugs and Crime, over 275 million people used drugs globally in 2020, resulting in 36 million individuals with drug use disorders. From 2010, there was a 22% increase in people who use drug, with an 11% increase projected by 2030 [1]. The National Anti-Drug Agency Malaysia arrested 20,643 people addicted to drugs in 2020, showing a decrease of 20.8% from 2019. The agency provides information on awareness programs, medicine, intervention, and rehabilitation, creating hot spots for drug abuse activities. In 2020, people who use and are addicted to drugs accounted for 2% of adolescents aged between 13 and 18 years. A total of 59.4% of all people who use drugs had a secondary education, but a recent study reported that approximately 5.5% of Malaysian youths are lifetime people who use drugs, which is relatively high compared with previous findings [2,3]. To combat the increasing number of adolescent people who use drugs in Malaysia, the government has focused on implementing measures to reduce drug use [4-6]. Drug abuse severely affects individuals, families, and society [7]. Consistent monitoring of drug abuse rates among Malaysian school-going youth is essential.

The need for information and statistics about people addicted to drugs, accessibility, usage, effects, and prevention is vital for reducing drug abuse [8]. However, local communities lack effective prevention tools despite drug abuse reaching epidemic levels [4,9]. The increasing drug use among school students contributes to the spread of drug abuse epidemics [10]. To address this challenge, geospatial analytics can provide location-based tools with statistical charts to assess prevalence and risk factors associated with substance abuse, aiding stakeholders’ understanding of the epidemic and promoting monitoring and prevention activities. Because of the substantial amount of assessment results that need to be compiled, it will be necessary to develop a digital system capable of managing, integrating, and synthesizing assessment data [11]. However, developing a user-friendly and effective dashboard can be a complex task that requires input from various stakeholders and careful consideration of user acceptance factors.

This paper is organized as follows. First, we provide a brief overview of the literature and related work. Next, we describe the methodology used in the study, including requirement gathering, pilot study, agile development, and data analysis. Then, we present the results of the study and discuss their implications. Finally, we conclude with suggestions for future research directions.

### Related Work

A digital dashboard tracks, analyzes, and manages information to indicate key performance related to a monitored subject matter [12,13]. However, sometimes a simple design is insufficient for understanding a specific topic that requires spatial analysis instead of simple visual inspection [14,15]. A geospatial dashboard provides a solution with a web-based interactive interface that combines mapping, spatial analysis, and visualization with dashboard functions [16]. In the case of drug abuse, geographical location needs to be considered to explore the location and distribution [17]. Though several dashboards are introduced in other sectors [18-20], few dashboards are found in the drug abuse sector.

As Muhamad et al [5] reported, psychoactive drugs have become a burden on public health globally, causing social difficulties and intentional overdose fatalities [21]. The government must focus on reducing teens who use drugs, and a geospatial dashboard can help through monitoring and prevention activities. However, previous dashboards were either agency-specific or not practical. This paper reports developing a geospatial dashboard for monitoring drug or substance abuse in Malaysian schools.

Several tools are available to help monitor and manage drug abuse, each with its unique strengths (see Multimedia Appendix 1) [8,9,21-30]. One such tool is DrugTracker [9], which combines social media and geographic data to detect and monitor drug abuse in near real time. This tool can help identify emerging drug trends and monitor drug abuse hot spots. Another tool, the Drug Abuse Information System [8], is designed to improve the storage and reporting of drug abuse information in Tanzania, making it easier to manage and prevent it. The Drug Abuse Information System is particularly useful for providing detailed data and reports on drug abuse, making tracking trends and identifying at-risk populations easier.

The VISN 22 dashboard [21] is another powerful tool to help detect veterans taking high-dose opioids and monitor and control for concomitant suicide risk factors in the United States. The dashboard helps review references to high-dose opioids prescribed with psychiatric illness and suicide risk factors. The Dashboard for Substance Abuse Prevention and Control [25] is a tool that uses survey data to help monitor, evaluate, and manage strategic performance in Los Angeles. It is beneficial for its ability to provide up-to-date raw data on patient age, employment, and criminal status risk factors, helping to identify at-risk populations and prevent drug abuse. Clinical Dashboards for Addiction Treatment [26] is another tool that arranges and presents patients’ data so clinicians can make informed, tailored medical decisions. This tool is valuable for its ability to provide detailed patient data as a pilot study in a Midwestern substance use disorder treatment center, allowing clinicians to tailor treatment to individual patients.

The Drug Control Data Dashboard [27] is a tool that allows users to search topics by year, agency, drug, and, to a limited degree, geographic location, providing a machine-readable and interactive collection of drug data from many sources in the United States. This tool is particularly useful for its ability to provide a wealth of data on many specific drug topics. The opioid-related adverse drug events dashboard [28] allows hospitals to access their opioid-related adverse drug events and local benchmark data against national trends in the United States. The visual analytics dashboard [29] is another tool that presents hospital-wide electronic health record medication alerts in Philadelphia to help reduce alert fatigue and improve medication safety. Finally, the Substance Abuse InstantAtlas dashboard [30] presents data on the health impacts of alcohol and selected drugs concerning public health in Alaska.

However, there are also limitations to these tools, such as incomplete and shallow social media data, lack of risk assessment information, and concerns about design flexibility and the ability to feed up-to-date raw data. Also, they have limitations regarding the scope of data, such as only reporting data on specific substance categories or a limited patient population. It is also important to note that some of the tools discussed in our study have not undergone user acceptance testing. Acceptance testing is an essential component for evaluating the user acceptance of any tool or product. Without proper acceptance testing, it is difficult to determine whether a tool is easy to use, useful, and effective in meeting the needs of its intended audience.

MyAsriGeo, a geospatial drug abuse risk assessment and monitoring dashboard tailored for school students, addresses the shortcomings of previous related works by offering unique features specifically designed to monitor drug use in Malaysian schools. In contrast to existing tools, it overcomes limitations in data comprehensiveness, depth of analysis, and spatial relevance by providing dynamic visualizations and seamless data connectivity. The dashboard focuses on the Malaysian school context, coupled with innovative features, and ensures a more comprehensive understanding of drug use patterns. It is validated using the Technology Acceptance Model (TAM), Post-Study System Usability Questionnaire (PSSUQ), and Task-Technology Fit (TTF) metrics. These assessments provided comprehensive insights into the ease of use, usefulness, interface quality, and sufficiency of MyAsriGeo. The process confirms that the geospatial dashboard transcends limitations, emerging as an innovative solution adept at meeting the diverse needs of users.

Overall, we provided a useful overview of different tools available for drug abuse detection and management, but it is important to carefully consider the strengths and limitations of each tool before selecting one for a specific use case.

## Methods

### Procedures

The methodology for the MyAsriGeo dashboard was based on agile prototyping and included iterative cycles of requirement analysis, dashboard design, development, and acceptance testing [31,32]. The use of the prototyping technique in the agile development process is a common practice among various studies and is highly favored by the agile community; this technique is likely well suited to be implemented in this type of process [33].

A student survey on adolescent drug abuse in Malaysia was conducted in collaboration with 85 schools, the Ministry of Education, Universiti Kebangsaan Malaysia, and the National Anti-Drugs Agency, comprising psychiatrists, psychologists, counselors, and experienced practitioners, through the MyAsri platform of the SEGAR project. It highlights around 3000 students to understand risk factors, prevalence, and elements of trajectories among adolescents involved in polydrugs and amphetamine-type stimulants abuse at selected hot spots in Malaysia. Drug abuse surveys offer a valuable understanding of the sociobehavioral dimensions related to adolescent drug abuse activities in Malaysia.

**Figure 1:**
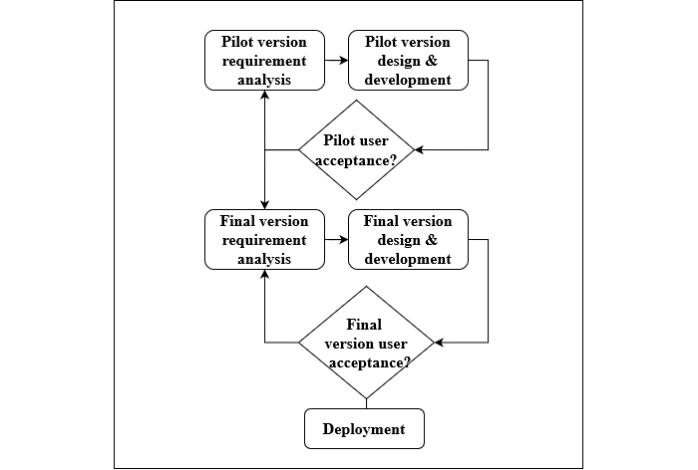
Methodology flowchart.

The MyAsriGeo dashboard underwent 2 rounds of requirement analysis and evaluation, as shown in Figure 1. Experts from different fields, including medical, research, development, education, geospatial, and drug rehabilitation domains, participated in 2 rounds of focus groups and interviews and 2 rounds of user acceptance.

### Ethical Considerations

With necessary approvals from relevant ethics committees (UKM PPl/111/8 JEP-2020-174(2)), parental consent was diligently obtained to ensure compliance with ethical standards. All data collected in this study were anonymized to protect the privacy and confidentiality of participants. Additionally, participants were compensated MYR RM20 (US $4.29) for their involvement in the research.

### Pilot Version Sprint

The pilot version of the MyAsriGeo geospatial drug abuse dashboard was designed and developed based on the basic requirements collected from the experts and stakeholders.

#### Pilot Version Requirement Analysis

The requirement analysis phase involved gathering comprehensive feedback from a diverse group of experts and stakeholders, including 20 experts spanning medical, research, development, education, geospatial, and drug rehabilitation domains. Aged between 30 and 59 years and possessing more than 5 years of experience in their respective fields, participants actively engaged in both focus group discussions and interviews, providing valuable insights. Thematic analysis was used during subsequent data analysis, systematically reviewing and coding detailed notes to identify recurring themes, patterns, and key insights, such as the selection of the contents of the results of the student surveys, demographics, notable trends in drug usage, specific challenges faced by students, and key points. This rigorous approach ensured a nuanced understanding of stakeholders’ perspectives. The resulting feedback served as a robust foundation for crafting the pilot version of the dashboard, strategically incorporating basic functionality to address critical needs identified in this collaborative and insightful process.

#### Pilot Version Design and Development

Based on the thematic analysis, the pilot version consisted of 3 pages, with no authentication or authorization system. The first page of the pilot version presented a basic view of the map, displaying the locations of schools without providing detailed summaries of key points. This page was designed to provide users with a simple and clear overview of the schools’ locations. The second and third pages of the pilot version were used to display the student survey results and the drug and substance abuse in the form of charts. These charts provided an overview of the prevalence of drug abuse among students, the types of drugs used, and other related information. The charts were designed to be visually appealing and easily interpreted, giving users a clear understanding of the survey results.

#### Pilot Version User Acceptance

The first TAM questionnaire was conducted for the 20 experts and stakeholders to evaluate the pilot version’s user acceptance such as ease of use and usefulness, and to identify areas for improvement. TAM is a theoretical framework used to predict how users will adopt and use technology. TAM suggests that usefulness and ease of use are important determinants of user acceptance and subsequent behavior. TAM has been widely used in various fields to analyze the adoption and usage of various technologies [22].

Initially, we crafted a survey using both a web and a paper-based questionnaire for individuals unable to access the online version. This survey aimed to explore the acceptance of the pilot version of the MyAsriGeo dashboard among the 20 experts and stakeholders, each with expertise in different specialty areas as mentioned earlier. The survey questions were designed, incorporating 5 items each for ease of use and usefulness (based on TAM). Responses were gathered using a Likert scale ranging from 1 to 5.

The feedback was used to refine the design and features of the dashboard. This process continued through multiple iterations until the MyAsriGeo dashboard was deemed effective in meeting the stakeholders’ needs and goals and could effectively support their efforts to address drug abuse in their communities.

### Final Version Sprint

#### Final Version Requirement Analysis

The feedback and acceptance results received from the experts and stakeholders who evaluated the pilot version were critical in identifying areas for improvement and ensuring that the final version of the dashboard met the needs of its intended users.

To define the end users and the new requirement for the MyAsriGeo dashboard, we conducted another focus group discussion and interviewed the experts and the stakeholders with our research team. During these sessions, we discussed the various demographic groups using the dashboard, including school-going youth, educators, and health care professionals. We also discussed the importance of including a variety of data and information on the dashboard, such as students’ demography and the Mooney Problem Checklist [34]; information on drug and substance abuse; and the Alcohol, Smoking, and Substance Involvement Screening Test (ASSIST) [35].

#### Final Version Design and Development

Based on the thematic analysis of the second requirement analysis, the results were presented in the dashboard, which were crucial in providing a comprehensive understanding of the drug abuse problem and helping users make informed decisions. Additionally, experts were concerned about the importance of including information on economic affordability and types of drugs being used and data on the time and place of drug use. These factors are considered in designing the MyAsriGeo dashboard to ensure that it effectively addresses the issue of drug abuse among students. The dashboard design phase focused on refining the pilot version through iterative feedback cycles, testing, and refinement. The development phase involved implementing the design and building the dashboard features, with each iteration building upon the previous one.

#### MyAsriGeo Architecture

**Figure 2:**
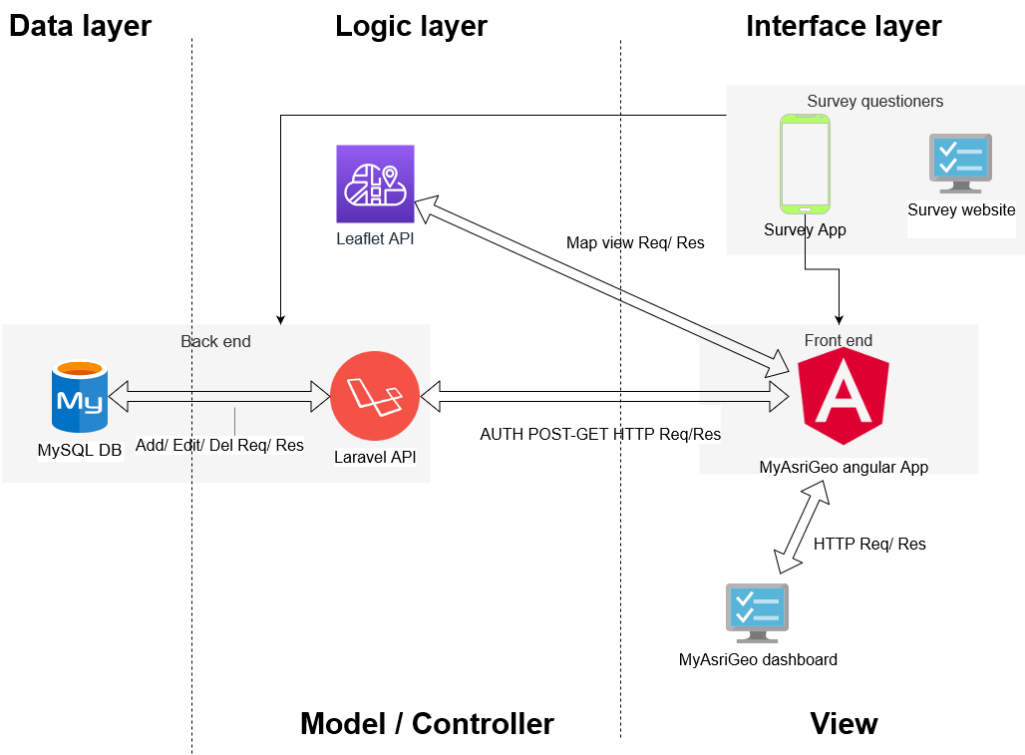
Architecture design of MyAsriGeo. API: application programming interface; DB: database.

The MyAsriGeo geospatial dashboard was developed using the Angular framework and TypeScript for the front end, with the Leaflet application programming interface (API) used for geomapping locations (as shown in Figure 2). The dashboard includes in-app charts to display students’ survey responses collected from mobile app surveys (which is not in the scope of this study), and aggregated data are stored in a MySQL database shared between survey applications and the Laravel API. The dashboard has a 3-tiered architecture, including the data, logic, and interface layers. The data layer holds geospatial and nongeographical data, and the logical layer contains models mapped to corresponding database tables in an object-oriented manner. The interface layer interacts directly with the client, communicating every client interaction with the logical layer containing the Leaflet API and Laravel API.

In crafting the MyAsriGeo architecture, our intentional choice to develop it as a web system is grounded in a forward-thinking approach, prioritizing future collaboration and sustainability. The incorporation of an API serves a dual purpose—facilitating seamless integration with external partners for potential collaborations and ensuring adaptability for future initiatives. The use of free services is pivotal for the project’s sustainability, providing scalability and maintenance without imposing significant financial constraints. Furthermore, MyAsriGeo’s tailored design specifically caters to the unique needs of drug abuse risk assessment for educational contexts, distinguishing it from other solutions. This strategic architectural approach positions MyAsriGeo as a versatile and enduring solution, adept at meeting the evolving demands of current and prospective stakeholders within the targeted field.

#### Final Version User Acceptance

Finally, the second Acceptance questionnaire was conducted to evaluate the final version of the dashboard; 2 additional factors added based on the PSSUQ and TTF models were enlisted to assess the interface quality and dashboard sufficiency for the final version by end-users and stakeholders. PSSUQ is a widely used measure of the usability of a system [24], while TTF measures the extent to which technology helps users perform their tasks effectively and efficiently [23].

Similar to the pilot version, we initiated the user acceptance for the final version of MyAsriGeo with the aforementioned experts. The survey questions were designed incorporating 5 items each for ease of use and usefulness (based on TAM), 4 items for interface quality (from PSSUQ), and 4 items for sufficiency (from TTF). Responses were gathered using a Likert scale ranging from 1 to 5. Subsequently, we presented the finalized dashboard to the aforementioned 20 experts and stakeholders. They were instructed to interact with and test the dashboard. After this hands-on experience, we administered the designed questionnaire to gather their feedback and insights.

## Results

### Requirement Analysis

The student survey application had 4 contents: demography, Mooney Problem Checklist, information on drug and substance abuse, and ASSIST. The first content, demography, gives an overview of the student’s life, including name, age, area, school, race, gender, religion, and economic status. The second content, the Mooney Problem Checklist [34], helps adolescents and adults to express personal difficulties, which is useful in enhancing teachers’ understanding of pupils and preparing children for counseling interviews. The topics of the checklist vary according to age group, health and physical development, home and family, morals and religion, romance, gender, and marriage. The third content, information on drug and substance abuse, identifies the information regarding drug and substance use, history, occurrence use, drug source, budget for drugs, medical history, prison history, abuse friends, and drug influencers of students. Finally, a multinational group of drug abuse researchers has created the World Health Organization’s ASSIST to screen for psychoactive substance use and associated problems in primary care patients [35]. ASSIST was considered the fourth content of the survey. The risk and its representation on the geospatial map are the indicators with the result of the previous survey that will give a big picture of the whole drug abuse information.

Textbox 1 outlines the components of the initial version of the dashboard, incorporating selected results from a student survey conducted. This pilot version primarily includes demographic information, providing a comprehensive overview of students’ demography, encompassing details such as name, age, area, school, race, gender, religion, and economic status. Additionally, it features information on drug and substance abuse, offering insights into students’ history, occurrence of use, drug sources, budget for drugs, medical history, prison history, associations with friends who abuse substances, and key influencers in their drug-related decisions.

**Contents of the results of the student survey, which were presented in the pilot version of the dashboard.**

1. Demography: Demographic information of the students.
2. Information on drug and substance abuse: Identify the information regarding drug and substance use of students.

Textbox 2 expands on the content integrated into the final version of the dashboard, building upon the initial selection. Based on the results of the second round of the focus group and interviews, additional features were incorporated. In addition to demographic data and information on drug and substance abuse, the final version includes the Mooney Problem Checklist [34] and the ASSIST tool. The Mooney Problem Checklist serves as a valuable tool to explore the problems students have experienced over the years, aiding in teachers’ understanding of students and facilitating counseling interviews. The ASSIST tool, designed by a multinational group of drug abuse researchers, is used to screen for psychoactive substance use and related problems in primary care patients [35]. These enhancements contribute to a more holistic understanding of the challenges faced by students, offering decision makers a comprehensive view of effective planning and intervention strategies. The inclusion of risk indicators and their representation on a geospatial map further enhances the dashboard’s capacity to provide a comprehensive picture of drug abuse patterns, enabling informed decision-making based on the survey results.

**Contents of the results of the student survey, which were presented in the final version of the dashboard.**

1. Demography: Demographic information of the students.
2. Mooney Problem Checklist [34]: Investigate the problems that students have experienced over the years.
3. Information on drug and substance abuse: Identify the information regarding drug and substance use of students.
4. The Alcohol, Smoking, and Substance Involvement Screening Test (ASSIST) [35]: It finds out the prohibited substances used by students in the last 3 months.

After the thematic analysis of the requirement analysis from the focus group discussion and interviews, we identified the key users for the MyAsriGeo dashboard who will use the dashboard (Textbox 3).

**The key users for the MyAsriGeo dashboard.**

1. Researchers benefit from the ability to access and analyze data on drug abuse patterns and trends in schools, which is crucial in informing their research and studies on the subject.
2. Police: The dashboard allows them to identify hot spot schools concerning drug abuse and crime, aiding their investigations and targeted enforcement efforts.
3. National Anti-Drugs Agency: They can access detailed information on hot spot areas, schools, and nearby amenities through the dashboard to assess risk and evaluate the effectiveness of student intervention programs.
4. Community: By accessing information on drug abuse patterns and trends, they can increase their awareness and aid in prevention efforts.
5. Teachers: The dashboard provides access to information on drug abuse patterns and trends in their schools, which can increase their awareness and aid in prevention efforts.
6. Administrators: The dashboard gives them the ability to access, control, and manage confidential data, assign roles, and manage accounts, which ensures data security and compliance.
7. Decision makers: By accessing data and analyzing drug abuse patterns and trends, they can inform and support decision-making to minimize risk, increase awareness, and minimize crime rates.

### MyAsriGeo Dashboard

**Figure 3:**
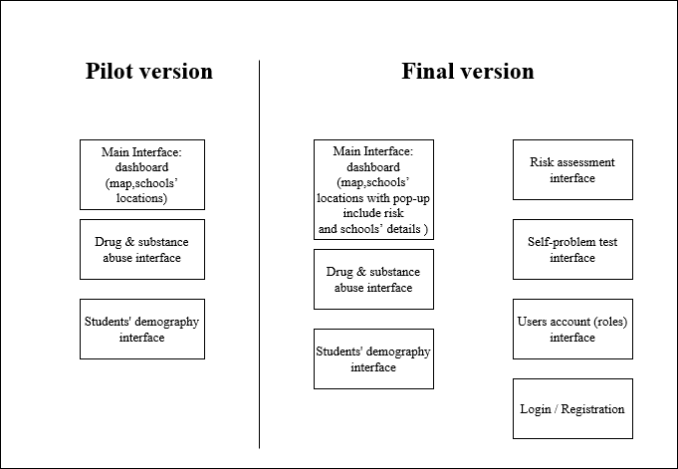
Pilot versus final version comparison.

The web application is structured with 7 distinct interfaces to effectively represent and interact with the collected data. In the pilot version (as shown in Figure 3), 3 primary interfaces are featured: the dashboard main page, providing an overarching view of key information with a basic map of the hot spot schools; the drug and substance use page, focusing on details related to substance use among students; and the students page, offering insights into the student population.

In the final version (as shown in Figure 3), the main page of the dashboard has been enhanced to include a dynamic map feature that provides detailed summaries of key points. This addition augments the visual representation of the data, allowing users to geospatially explore and analyze critical information. Additional interfaces are introduced to enhance the functionality of the application. The risk assessment page provides a detailed breakdown of drug abuse risks, allowing for a more nuanced understanding of specific challenges. The self-problem test page facilitates self-assessment for students, aiding in the identification of personal difficulties and contributing to a proactive approach to addressing concerns. The users page and authentication interface are incorporated to manage user access and permissions securely, ensuring the confidentiality and integrity of the data.

The synchronization of information from the main database back to the geospatial web across these interfaces strengthens the application’s ability to provide a comprehensive and accessible representation of the survey results. Together, these interfaces empower users with the tools they need to analyze, understand, and respond effectively to the collected data, fostering informed decision-making and intervention planning.

#### Main Interface: Dashboard

**Figure 4:**
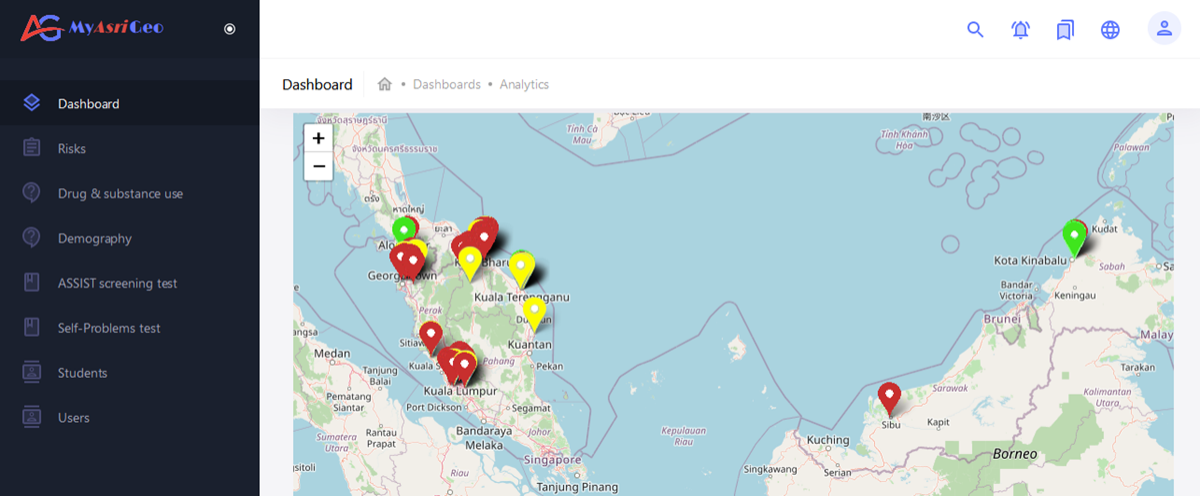
The main interface design of the MyAsriGeo dashboard.

The MyAsriGeo dashboard has a main interface (as shown in Figure 4) with a menu bar containing 7 options for easy navigation. The interface also displays a geolocation map of hot spot schools in Malaysia with markers colored red where at least one of the students is at a high risk for drug abuse, yellow where at least one of the students is at a medium risk, or green for otherwise. The pop-up for each school provides general statistics such as school name, student count, and school risk. The interface also includes a drop-down list to filter results by drug type. The school risk equation is displayed based on equation 1:

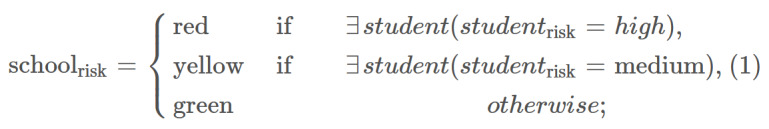



#### Risk Assessment Interface

The risk assessment page displays individual risks for different types of substance abuse in pie charts, which can be filtered by state and school using drop-down menus. The platform uses the ASSIST screening tool to assess the severity of substance abuse and assigns a numerical score to each question [35], which is then used to determine a low-, medium-, or high-risk level for each substance. Various factors such as family history and socioeconomic level are considered when assessing a student’s risk of substance abuse; we represent the calculation of student risk as follows [35]:

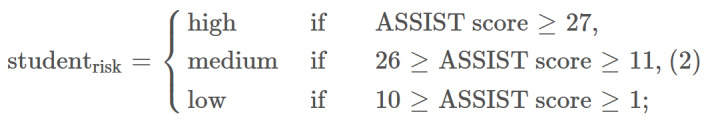



#### Drug and Substance Abuse Interface

**Figure 5:**
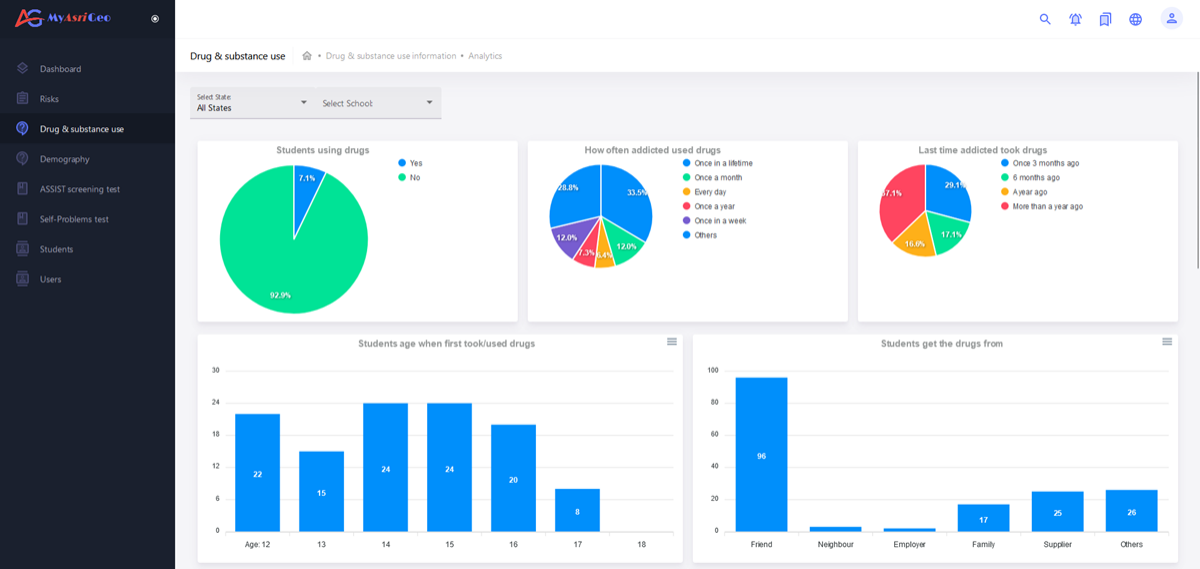
The drug and substance use interface of MyAsriGeo.

The drug and substance abuse page of MyAsriGeo (as shown in Figure 5) displays charts related to drug and substance use, including students’ age, gender, medical history, parental marital status, financial status, frequency of drug use, age of first drug use, source of drugs, expenses of drug use, hospitalizations and imprisonment due to drug use, and drug-addicted friends. Users can filter the charts by state and school using drop-down lists. Pie charts and bar charts represent the data whereby the user can scroll down to view more charts.

#### Self-Problem Test Interface

This page presents the Mooney self-problem test result in bar charts with 2 state and school selection filters. Around 60 charts are presented here to cover the questions about money, communication, life, family, faith, job, school, future, and learning. The answers are categorized based on the options (strongly agree, agree, disagree, do not agree, and very not agree) given during survey data collection. The charts can be exported to CSV files, PNG images, and SVG vector-based images.

#### Students’ Demography Interface

**Figure 6:**
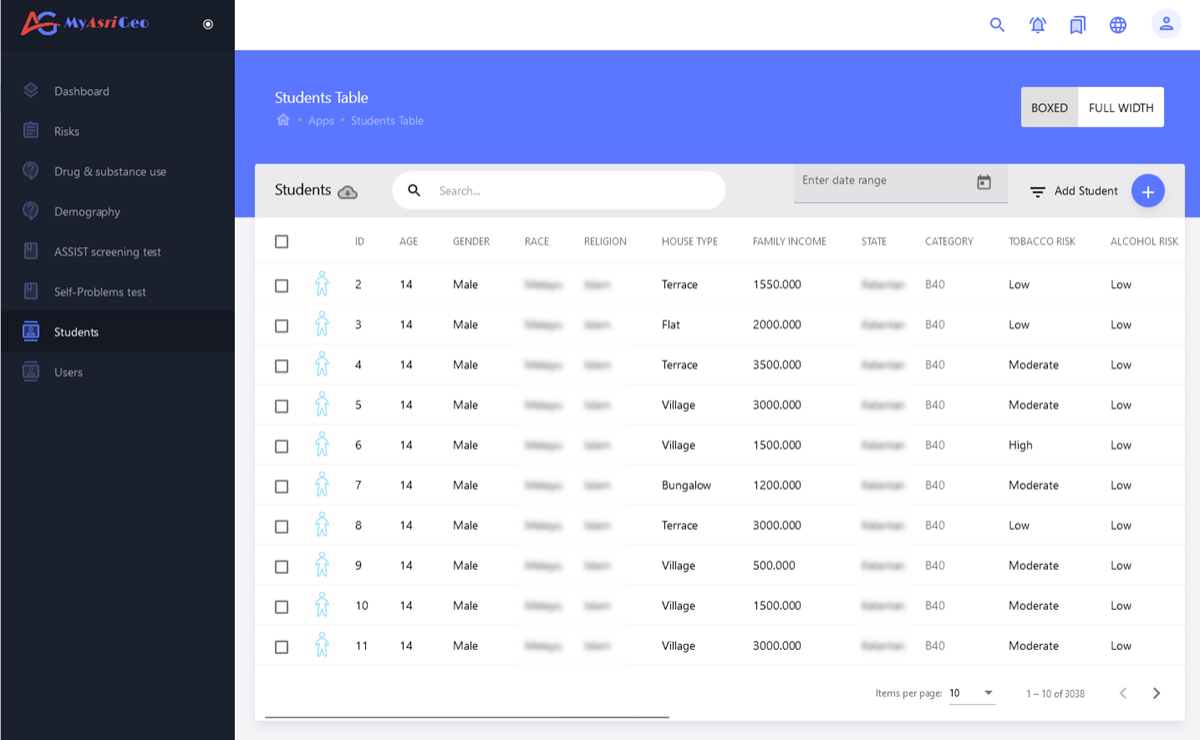
Students page of MyAsriGeo.

On this page (as shown in Figure 6), students’ demographic information is listed in a table. Individual students’ risk levels for each drug type can be viewed on this page. Each row has an edit and delete button to edit and delete the student information. Apart from that, there is a button on the top of the table to search for any field type. The table can be sorted column-wise for each field. Ascending and descending options, including eliminating field columns, are also available. Options such as exporting tables in Excel form and adding new students are available on this page.

#### Users Account Interface

The MyAsriGeo system has 3 main user roles: administrator, country access user, and state access user. The roles determine the level of access to information and privileges granted to each user; the administrator has all the privileges, including managing user accounts by giving access to newly created accounts and editing and deleting accounts. The country access user can access everything except managing user accounts, while the state access user can access state-level schools, students, risks, and other information. A confirmation email is sent to new account holders to verify their email, and the administrator will review the account and assign appropriate access based on their assigned role. The users’ information is listed in a table with add, edit, and delete buttons, search, and filters. The passport token scope allows users to access information based on their role, ensuring that only authorized users can access sensitive data.

### User Acceptance Results

Table 1 shows the statistical analysis results comparing the means of 2 factors (ease of use and usefulness) between the pilot version and the final version of a dashboard. For ease of use, the mean score was 3.77 in the pilot version and 4.2 in the final version. The 2-tailed *P* value of .047 indicates a statistically significant difference between the 2 means. The *T* value of –2.1 shows the direction and magnitude of the difference. The effect size, measured by partial η^2^, was 0.191, indicating a large effect size. This suggests that the changes made to the dashboard substantially impacted users’ perception of its ease of use.

**Table 1:** Summary of the user acceptance results after the pilot prototype and the final version (N=20).

Factor	Pilot prototype, mean (SD)	Final version, mean (SD)	2-tailed *P* value	*T* value *(df)*	η^a^
Ease of use	3.77 (0.93)	4.2 (0.55)	.047	–2.1 (19)	0.191
Usefulness	3.9 (1.09)	4.46 (0.43)	.02	–2.5 (19)	0.248
Interface quality	—^a^	4.29 (0.49)	—	—	—
Sufficiency	—^a^	4.13 (0.65)	—	—	—

^a^Not applicable (these 2 factors were only designed for the final version evaluation).

For usefulness, the mean score was 3.9 in the pilot version and 4.46 in the final version. The 2-tailed *P* value of .02 indicates a statistically significant difference between the 2 means. The *T* value of –2.5 shows the direction and magnitude of the difference. The effect size, measured by partial η^2^, was 0.248, indicating a large effect size. This suggests that the changes made to the dashboard substantially impacted users’ perception of its usefulness.

The table shows the mean and SD scores for 2 different measures of user interface quality [24] and sufficiency [23]. PSSUQ is a widely used measure of the usability of a system, while TTF measures the extent to which technology helps users perform their tasks effectively and efficiently. The mean score for interface quality on PSSUQ was 4.29, indicating that users generally found the interface to be of high quality. The SD for PSSUQ was 0.49, indicating that the ratings were relatively consistent. The mean score for sufficiency on TTF was 4.13, indicating that users generally found the system sufficient for their needs. The SD for TTF was slightly higher at 0.65, indicating that ratings were slightly more varied.

To conclude, these results suggest that the changes made to the dashboard between the pilot and final versions significantly impacted users’ perceptions of its ease of use and usefulness. The effect size was large for ease of use and large for usefulness, indicating that the changes were meaningful and had a noticeable impact on users’ experience with the dashboard. The scores for interface quality and sufficiency of the final version show that users are satisfied with the quality of the MyAsriGeo interface and have found its content sufficient.

## Discussion

### Principal Findings

Dashboards have become a popular tool in various health care systems to assist in making informed decisions about patient care by presenting a vast amount of information concisely and easily comprehensibly. Their implementation is on the rise as they enhance the quality and safety of care while reducing drug abuse [36,37]. The MyAsriGeo geospatial dashboard, developed to handle and analyze multivariate students’ data regarding drug usage, is a valuable resource for stakeholders in Malaysia to understand and respond to the issue of drug abuse among youth. The development process involved continuous feedback from experts and stakeholders, ensuring that the dashboard was designed to meet the needs of all user types. The acceptance evaluation revealed that the dashboard was user-friendly and useful, indicating that it can be a valuable resource for stakeholders involved in addressing the issue of drug abuse among youth.

The high level of interface quality and sufficiency achieved by the MyAsriGeo dashboard is an important outcome, as a well-designed and well-functioning dashboard can greatly enhance the quality and usefulness of the tool [23]. By providing a geospatial map of high-risk areas, data on drug abuse among students, and risk assessment tools, the dashboard helps stakeholders understand the extent of the problem, identify areas of concern, and make informed decisions to address the issue.

The involvement of various stakeholders in developing and evaluating the MyAsriGeo dashboard is also noteworthy. By gathering the feedback and requirements of the National Anti-Drugs Agency, police, schoolteachers, students, and researchers, the dashboard was designed to meet the diverse needs of the users. It enhances the acceptance and adoption of the tool, as it is tailored to the specific needs of the stakeholders.

The evaluation results of the MyAsriGeo dashboard with experts and stakeholders suggest that the changes made to the dashboard between the initial prototype and the final version significantly impacted users’ perceptions of its ease of use and usefulness [22]. Specifically, the results indicate that changes to the dashboard greatly affected users’ perception of its ease of use and usefulness. The use of agile methodology and prototyping in the design and development of the dashboard allows for close collaboration with experts and stakeholders throughout the development process. This iterative approach facilitated identifying and incorporating feedback and requirements from these key stakeholders [38], which likely contributed to the dashboard’s success.

The TAM, PSSUQ, and TTF were used to assess user acceptance of the dashboard. The TAM enabled the evaluation of key aspects of the dashboard’s acceptance and provided insight into users’ attitudes toward the dashboard. The involvement of 20 experts in evaluating the dashboard further supports the reliability and validity of the findings.

The dashboard supports enhancing decision-making abilities of decision makers, providing insights not only on which drugs are abused by students in hot spot locations but also on the impact those drugs have on the students’ resulting health risk. Thus, it allows the decision maker to get precise information and use it accordingly [39].

The results of this study suggest that the MyAsriGeo dashboard, designed and developed using agile methodology and prototyping, is a promising tool for monitoring and analyzing drug abuse trends. The significant impact of the changes made to the dashboard between the initial prototype and final version on users’ perceptions of its ease of use and usefulness highlights the importance of ongoing evaluation and refinement of such tools in collaboration with key stakeholders.

To ensure the effectiveness of the MyAsriGeo dashboard, continuous updates and improvements are necessary to keep up with the changing landscape of drug abuse among students. As such, future research can explore including more data sources and real-time data analysis to enhance the accuracy and effectiveness of the dashboard. It can include integrating social media data or information from drug rehabilitation centers.

The review highlighted several limitations of the tools, including incomplete and shallow social media data, lack of risk assessment information, concerns about design flexibility, and the ability to feed up-to-date raw data, and limitations regarding the scope of data they cover. Additionally, some tools have not undergone user acceptance, which is crucial in evaluating the effectiveness of any tool or product [21,25-30].

Therefore, before selecting a tool for a specific use case, it is important to consider its strengths and limitations carefully. The review provided a useful overview of different tools available for drug abuse detection and management, but it is important to understand that no tool is perfect, and each has its own set of limitations. It is also crucial to conduct user acceptance to ensure that a tool is user-friendly, efficient, and effective in meeting the needs of its intended audience. Overall, the discussion emphasizes the need for a careful and critical evaluation of the available tools to make informed decisions about their use.

Additionally, the MyAsriGeo dashboard can be extended to cover other areas beyond drug abuse, such as cyberbullying, mental health issues, and academic performance. It can help stakeholders to identify potential risk factors that may contribute to drug abuse among students and take preventive measures.

It is important to note that the MyAsriGeo dashboard is not a stand-alone solution for addressing drug abuse among youth. A comprehensive strategy must complement it, including education, prevention, treatment, and law enforcement efforts [40]. The dashboard can provide stakeholders with the necessary information and insights to make informed decisions, but it is up to them to take action and address the issue of drug abuse among youth.

### Limitations

The involvement of 20 experts in the evaluation provides some evidence for the reliability and validity of the findings; a larger sample size would strengthen the generalizability of the results. Additionally, the study was limited to evaluating the dashboard’s user acceptance, and future research could explore the impact of the dashboard on actual decision-making processes and outcomes.

### Future Work

Future research could explore the effectiveness of the MyAsriGeo dashboard in detecting and monitoring drug abuse trends over time. Longitudinal studies could assess whether the dashboard provides decision makers with accurate and timely information to identify and respond to emerging drug abuse patterns. Additionally, future work could investigate the potential for the dashboard to be adapted and implemented in different contexts, such as other regions or countries, to assess its scalability and generalizability. Finally, integrating additional data sources, such as social media, could enhance the dashboard’s ability to provide decision makers with a comprehensive view of drug abuse trends.

### Conclusions

The geospatial drug abuse dashboard used in this study was developed and evaluated with the help of experts and stakeholders. The results showed that the changes made between the initial and final versions of the dashboard significantly impacted users’ perceptions of its ease of use and usefulness. This study demonstrates the importance of geospatial dashboards for drug abuse monitoring and analysis among students.

## Appendix

Multimedia Appendix 1 Evaluation questionnaire and related work comparison.

## Abbreviations

API: application programming interface
ASSIST: Alcohol, Smoking, and Substance Involvement Screening Test
PSSUQ: Post-Study System Usability Questionnaire
TAM: Technology Acceptance Model
TTF: Task-Technology Fit

## References

[1] (2021). World Drug Report 2021: pandemic effects ramp up drug risks, as youth underestimate cannabis dangers. United Nations Office on Drugs and Crime.

[2] Ismail R, Manaf MRA, Hassan MR, Nawi AM, Ibrahim N, Lyndon N, Amit N, Zakaria E, Razak MAA, Nor NIZ, Shukor MS, Kamarubahrin AF (2022). Prevalence of drug and substance use among malaysian youth: a nationwide survey. Int J Environ Res Public Health.

[3] (2019). National Health and Morbidity Survey (NHMS) 2019: non-communicable diseases, healthcare demand, and health literacy-key findings. Institute for Public Health.

[4] (2020). Drug and substance abusers and addicts. National Anti-Drug Agency.

[5] Muhamad NA, Mihat O, Ramly R, Aziz AA, Kamaruddin R, Mansor WNAW, Abdullah NH, Noor MAM, Ismail R, Wisman WA, Lodz NA, Yusoff MFM (2018). Translation, cross-cultural adaptation, reliability and validity of the malay version of alcohol, smoking and substance involvement screening test (ASSIST) V3.1. Health.

[6] Drus ZAM, Singh D, Mokhtar MR, Rashid RA (2018). Review of computerized cognitive behavioural therapy based on culture centered design for substance abuse in Malaysia. Asia Pac J Inf Technol Multimed.

[7] Eric P (2017). Socioeconomic effects of drug abuse among Nigerian youths. Can Sci Med.

[8] Mnunguli JP (2019). Development of a web and mobile application for drug abuse information awareness. Nelson Mandela African Institution of Science and Technology.

[9] Hu H, Phan N, Ye X, Jin R, Ding K, Dou D (2019). DrugTracker: a community-focused drug abuse monitoring and supporting system using social media and geospatial data (Demo Paper). https://dl.acm.org/doi/10.1145/3347146.3359076.

[10] Chie QT, Tam CL, Bonn G, Wong CP, Dang HM, Khairuddin R (2015). Drug abuse, relapse, and prevention education in Malaysia: perspective of university students through a mixed methods approach. Front Psychiatry.

[11] Hsiao CT, Chou FC, Hsieh CC, Chang LC, Hsu CM (2020). Developing a competency-based learning and assessment system for residency training: analysis study of user requirements and acceptance. J Med Internet Res.

[12] Liu S (2021). Design and implementation of a geospatial dashboard for crime analysis and prediction. Toronto Metropolitan University.

[13] Few S (2006). Information Dashboard Design: The Effective Visual Communication of Data.

[14] Abdullah MA, Abdullah SNHS, Nordin MJ (2018). Additional feet-on-the-street deployment method for indexed crime prevention initiative. J Pengur.

[15] Alzahrani NA, Abdullah SNHS, Mohamed I, Mukred M (2021). The adoption of geographic information systems in the public sector of Saudi Arabia: a conceptual model. Math Probl Eng.

[16] Jing C, Du M, Li S, Liu S (2019). Geospatial dashboards for monitoring smart city performance. Sustainability.

[17] Ghazvini A, Abdullah SNHS, Hasan MK, Bin Kasim DZA (2020). Crime spatiotemporal prediction with fused objective function in time delay neural network. IEEE Access.

[18] Rochin MAE, Gutierrez-Garcia JO, Rosales JJ, Rodriguez LF (2021). Design and evaluation of a dashboard to support the comprehension of the progression of patients with dementia in day centers. Int J Med Inform.

[19] Niu Y, Ying L, Yang J, Bao M, Sivaparthipan CB (2021). Organizational business intelligence and decision making using big data analytics. Inf Process Manag.

[20] Yang J, Xiu P, Sun L, Ying L, Muthu B (2022). Social media data analytics for business decision making system to competitive analysis. Inf Process Manag.

[21] Kryskalla J, Kern S, Gray D, Hauser P (2014). Using dashboard technology to monitor overdose risk. Fed Pract.

[22] Davis F (1987). User acceptance of information systems: the technology acceptance model (TAM). Graduate School of Business.

[23] De Wulf K, Schillewaert N, Muylle S, Rangarajan D (2006). The role of pleasure in web site success. Inf Manag.

[24] Lewis JR (1995). IBM computer usability satisfaction questionnaires: psychometric evaluation and instructions for use. Int J Hum Comput.

[25] Alibrahim A, Wu S, Guerrero E (2014). Performance dashboard for the substance abuse treatment system in Los Angeles.

[26] Wolf DAPS, BlackDeer AA, Beeler-Stinn S, Zheng K, Stazrad K (2020). Performance-based practice: clinical dashboards for addiction treatment retention. Res Social Work Prac.

[27] Tableau Software (2022). Office of national drug control policy, drug control data dashboard. The White House.

[28] Stone AB, Jones MR, Rao N, Urman RD (2019). A dashboard for monitoring opioid-related adverse drug events following surgery using a national administrative database. Am J Med Qual.

[29] Simpao AF, Ahumada LM, Desai BR, Bonafide CP, Gálvez JA, Rehman MA, Jawad AF, Palma KL, Shelov ED (2015). Optimization of drug-drug interaction alert rules in a pediatric hospital's electronic health record system using a visual analytics dashboard. J Am Med Inform Assoc.

[30] Coughlin L, Hull-Jilly D, Saxon S (2019). Creating an instantatlas™ dashboard for Alaska substance use and abuse data.

[31] Cohen D, Lindvall M, Costa P (2004). An introduction to agile methods. Advances in Computers: Advances in Software Engineering.

[32] Budde R, Kautz K, Kuhlenkamp K, Züllighoven H (1990). What is prototyping?. Inf Technol People.

[33] de Oliveira Sousa A, Valentim NMC (2019). Prototyping usability and user experience: a simple technique to agile teams. https://dl.acm.org/doi/abs/10.1145/3364641.3364667.

[34] Mooney RL, Gordon LV (1950). Mooney problem check lists. PsycTESTS Dataset.

[35] Humeniuk R, Henry-Edwards S, Ali R, Poznyak V, Monteiro MG (2010). The Alcohol, Smoking and Substance Involvement Screening Test (ASSIST): manual for use in primary care. World Health Organization.

[36] Dowding D, Randell R, Gardner P, Fitzpatrick G, Dykes P, Favela J, Hamer S, Whitewood-Moores Z, Hardiker N, Borycki E, Currie L (2015). Dashboards for improving patient care: review of the literature. Int J Med Inform.

[37] Buttigieg SC, Pace A, Rathert C (2017). Hospital performance dashboards: a literature review. J Health Organ Manag.

[38] Cao L, Ramesh B (2008). Agile requirements engineering practices: an empirical study. IEEE Softw.

[39] Wu E, Villani J, Davis A, Fareed N, Harris DR, Huerta TR, LaRochelle MR, Miller CC, Oga EA (2020). Community dashboards to support data-informed decision-making in the HEALing communities study. Drug Alcohol Depend.

[40] Ismail Rozmi, Shafurdin Nurul Shafini, Shukor Md Shafiin, Mohammed Nawi Azmawati, Abdul Manaf Mohd Rizal, Ibrahim Norhayati, Mohd Rasdi Roziah, Lyndon Novel Anak, Amit Noh, Hassan Siti Aishah, Hanafi Norshafizah, Ibrahim Fauziah, Nahla Fathimath, Wahab Suzaily (2024). Predictors of drug and substance abuse among school-going adolescents living in drug hotspot in Malaysia. PLoS One.

