# Supplementary material for: A Geospatial Drug Abuse Risk Assessment and Monitoring Dashboard Tailored for School Students: Development Study With Requirement Analysis and Acceptance Evaluation
Source: JMIR Hum Factors. 2024 Jul 30;11:e48139. doi: 10.2196/48139 (PMC11322689; doi:10.2196/48139)
Supplement: Multimedia Appendix 1 [file humanfactors_v11i1e48139_app1.pdf]

Multimedia Appendix

Table S1. Summary of the evaluation questionnaire.

Factor	Questions	Reference
Ease of Use	How easy is the MyAsriGeo dashboard to use?	TAM [22]
	It is easy to use	
	It can be used without referring to a user manual	
	It is flexible to interact with	
	It is easy to get information using MyAsriGeo to do what I want to do	
	It is easy to detect and correct errors in data records	
Usefulness	How useful is the MyAsriGeo dashboard?	TAM [22]
	Is capable of enhancing work effectiveness.	
	It will increase users' productivity at work.	
	It will enable users to accomplish tasks more quickly.	
	This will enable users to accomplish tasks more efficiently.	
	Provides greater control over the user's work	
User interface quality	How useful and easy is the MyAsriGeo user Interface?	PSSUQ [24]
	MyAsriGeo dashboard has all the capabilities I expected it to have.	
	I like using the interface of this system	
	The interface of MyAsriGeo is pleasant.	
	overall, I am satisfied with MyAsriGeo dashboard.	
Sufficiency	The web site offers enough information.	TTF [23]
	I find a satisfactory amount of information in the web site.	
	The web site provides sufficient information	
	I do not feel I miss information in the web site.	

Figure S1. Strengths/limitations comparison of related work figure

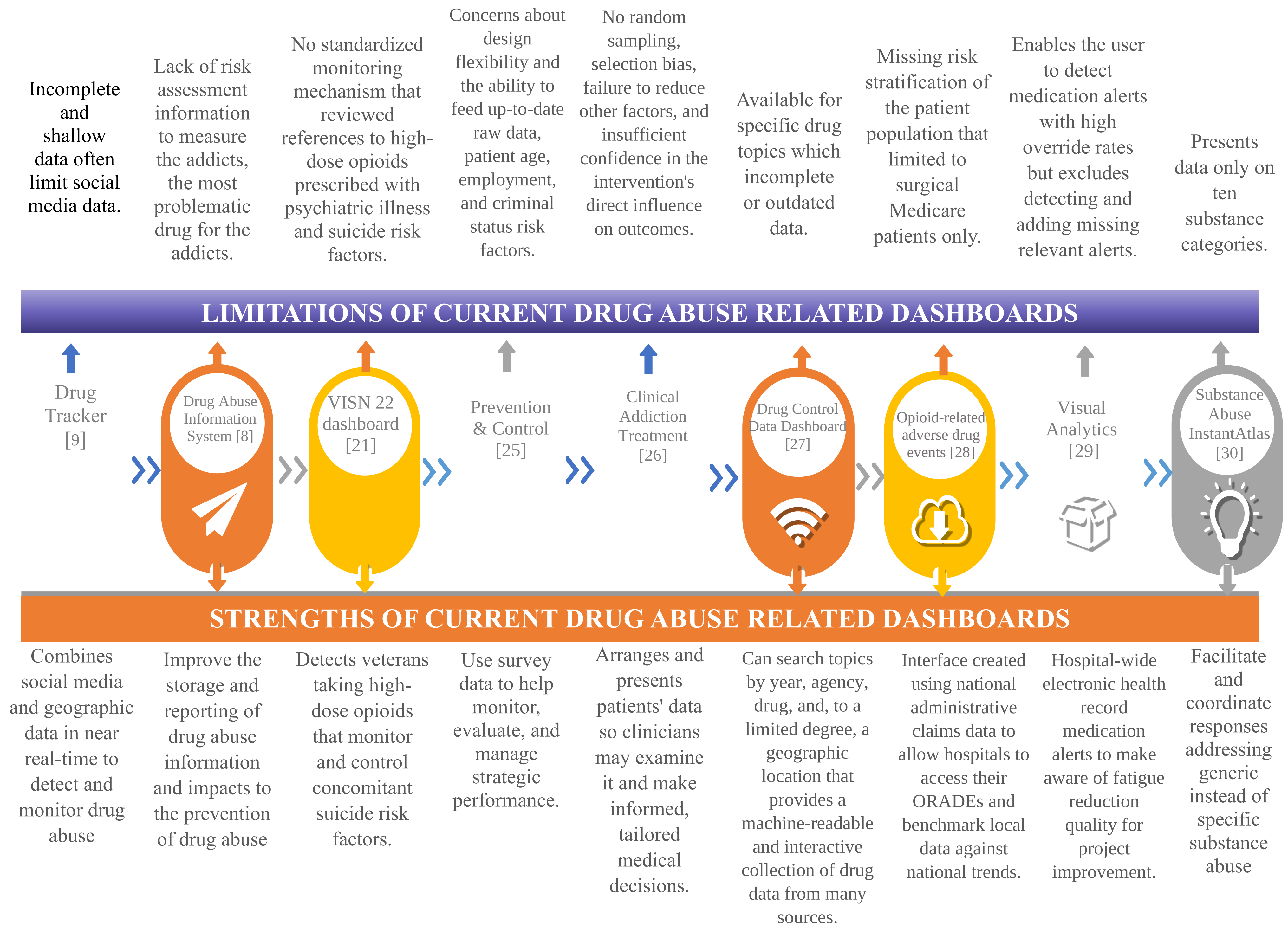
LIMITATIONS OF CURRENT DRUG ABUSE RELATED DASHBOARDS
Incomplete and shallow data often limit social media data.
Lack of risk assessment information to measure the addicts, the most problematic drug for the addicts.
No standardized monitoring mechanism that reviewed references to high-dose opioids prescribed with psychiatric illness and suicide risk factors.
Concerns about design flexibility and the ability to feed up-to-date raw data, patient age, employment, and criminal status risk factors.
No random sampling, selection bias, failure to reduce other factors, and insufficient confidence in the intervention's direct influence on outcomes.
Available for specific drug topics which incomplete or outdated data.
Missing risk stratification of the patient population that limited to surgical Medicare patients only.
Enables the user to detect medication alerts with high override rates but excludes detecting and adding missing relevant alerts.
Presents data only on ten substance categories.
Drug Tracker [9]
Drug Abuse Information System [8]
VISN 22 dashboard [21]
Prevention & Control [25]
Clinical Addiction Treatment [26]
Drug Control Data Dashboard [27]
Opioid-related adverse drug events [28]
Visual Analytics [29]
Substance Abuse InstantAtlas [30]
STRENGTHS OF CURRENT DRUG ABUSE RELATED DASHBOARDS
Combines social media and geographic data in near real-time to detect and monitor drug abuse
Improve the storage and reporting of drug abuse information and impacts to the prevention of drug abuse
Detects veterans taking high-dose opioids that monitor and control concomitant suicide risk factors.
Use survey data to help monitor, evaluate, and manage strategic performance.
Arranges and presents patients' data so clinicians may examine it and make informed, tailored medical decisions.
Can search topics by year, agency, drug, and, to a limited degree, a geographic location that provides a machine-readable and interactive collection of drug data from many sources.
Interface created using national administrative claims data to allow hospitals to access their ORADEs and benchmark local data against national trends.
Hospital-wide electronic health record medication alerts to make aware of fatigue reduction quality for project improvement.
Facilitate and coordinate responses addressing generic instead of specific substance abuse

Table S2. Feature comparison table of the related work.

Features/tools	DrugTracker [9]	Drug Abuse Information System (DAIS) [8]	VISN 22 dashboard [21]	Dashboard for Substance Abuse Prevention and Control (SAPC) [25]	Clinical Dashboards for Addiction Treatment [26]	Drug Control Data Dashboard (DCDD) [27]	Opioid-related adverse drug events (ORADEs) Dashboard [28]	Visual Analytics Dashboard [29]	Substance Abuse InstantAtlas™ dashboard [30]	MyAsrigeo
Support risk map	N/A	×	N/A	×	×	N/A	×	×	×	✓
Support geospatial location	✓	✓	N/A	×	N/A	N/A	N/A	N/A	✓	✓
Support Risk	✓	×	✓	✓	N/A	N/A	N/A	N/A	N/A	✓
Support Demography	×	✓	N/A	✓	✓	N/A	✓	N/A	N/A	✓
Support a wide range of drugs types	N/A	✓	×	×	N/A	N/A	N/A	N/A	✓	✓
Support Access control	N/A	✓	N/A	×	N/A	N/A	N/A	✓	N/A	✓
Support Authentication	N/A	✓	N/A	×	N/A	N/A	N/A	✓	N/A	✓
Support temporal	✓	✓	N/A	✓	✓	N/A	✓	✓	✓	✓
Support problem test	×	×	N/A	×	✓	N/A	N/A	N/A	N/A	✓
Support Security	N/A	×	N/A	×	N/A	N/A	N/A	✓	✓	✓
Support Health indicators	N/A	✓	✓	✓	✓	✓	✓	N/A	✓	✓
Support Information on Drug Abuse	✓	✓	✓	✓	✓	✓	✓	✓	✓	✓
Support prediction	×	×	×	×	×	N/A	×	×	×	×
Support DataMining	✓	×	×	×	×	N/A	×	×	×	×
